# Comorbidities in Mycosis Fungoides and Racial Differences in Co-Existent Lymphomatoid Papulosis: A Cross-Sectional Study of 580 Patients in an Urban Tertiary Care Center

**DOI:** 10.3390/medicines7010001

**Published:** 2019-12-26

**Authors:** Subuhi Kaul, Micah Belzberg, John-Douglas Matthew Hughes, Varun Mahadevan, Raveena Khanna, Pegah R. Bakhshi, Michael S. Hong, Kyle A. Williams, Annie L. Grossberg, Shawn G. Kwatra, Ronald J. Sweren

**Affiliations:** 1 Department of Medicine, John H. Stroger Hospital of Cook County, Chicago, IL 60612, USA; 2 Department of Dermatology, Johns Hopkins University School of Medicine, Baltimore, MD 21287, USA; mbelzbe@jhu.edu (M.B.); vmahade1@jhu.edu (V.M.); rkhanna8@jhmi.edu (R.K.); pb746@georgetown.edu (P.R.B.); michael.hong@som.umaryland.edu (M.S.H.); kwill184@health.fau.edu (K.A.W.); agrossb2@jhmi.edu (A.L.G.); skwatra1@jhmi.edu (S.G.K.); rsweren1@jhmi.edu (R.J.S.); 3 Section of Dermatology, University of Calgary, Alberta, AB T2N 1N4, Canada; jhugh017@uottawa.ca

**Keywords:** mycosis fungoides, atopic dermatitis, psoriasis, associations, comorbidities, epidemiology, lymphomatoid papulosis, lymphoma, racial differences

## Abstract

**Background:** Mycosis fungoides (MF) is a cutaneous T-cell lymphoma. Previous reports have suggested MF is associated with inflammatory conditions such as psoriasis, increased cardiovascular risk factors as well as secondary neoplasms. **Methods:** A cross-sectional study of MF patients seen from 2013 to 2019 was performed. Comorbidities were selected based on the 2015 Medicare report highlighting the most common chronic medical illnesses in the United States. Lifetime comorbidity occurrence in patients with MF were compared with that in patients with atopic dermatitis, psoriasis and patients without MF. Additional analyses were performed with patients sub-stratified by race. **Results:** Compared to control groups, MF was strongly associated with lymphomatoid papulosis and Hodgkin’s disease, but not significantly associated with lung, breast or colon cancer. Interestingly, the association with lymphomatoid papulosis was observed in Caucasians (CI 1062-4338; *p* < 0.001) and not African Americans (*p* = 0.9). Patients with MF had a greater association with congestive heart failure, hypertension (HT) and hyperlipidemia (HLD) compared with the general population. However, they were significantly less likely to have HT and HLD when compared with psoriasis patients (HT CI: 0.6–0.9; *p* < 0.001, and HLD CI: 0.05–0.07; *p* < 0.001). MF patients were also significantly less likely to have concomitant vitamin D deficiency compared with atopic dermatitis (AD) and psoriasis (*p* < 0.001). **Conclusions:** Our results suggest that the association of MF with lymphomatoid papulosis varies by race. Compared to the general population, hypertension and hyperlipidemia were positively associated with MF, however, these were significantly less likely on comparison to psoriasis. Unlike previously described, vitamin D deficiency was found to be significantly less in patients with MF.

## 1. Introduction

Mycosis fungoides (MF) is the most common cutaneous T-cell lymphoma (CTCL), accounting for approximately half of all primary cutaneous lymphomas [1]. Although the incidence of CTCL was rising in the 1970s, (due to either a real increase in cases, improvement in diagnostic methods, or a combination of the two) it has since stabilized to 5.6 per million persons with MF in the United States [2,3,4]. 

While most commonly observed after the age of 55 years, MF onset can arise in early adulthood or childhood with a nearly 2:1 male to female ratio [2]. MF typically has a slow and progressive disease course with patches, plaques and tumors developing sequentially. However, nearly 30% of patients demonstrate erythroderma or skin tumors at the outset [2]. Advanced disease involving blood, lymph nodes and visceral organs occurs in close to a third of cases [1]. Moreover, patients with MF are reported to be at an increased risk of developing secondary neoplasms, particularly Hodgkin lymphoma and lymphomatoid papulosis [2].

Recent evidence points to a relationship with inflammatory disorders like psoriasis—attributable to similarities in pathogenesis and the possible role of Toll-like receptors in both [5,6]. Patients with MF experience increased rates of cardiovascular risk factors [7], and apart from those experiencing limited plaque/patch stage (T1) MF, lower overall survival when compared to healthy controls matched for race, age and sex [8,9]. Being a chronic relapsing disease, the presence of comorbid conditions can potentially add to patient burden.

Considering these new observations, further investigation to elucidate the comorbidities and risk of selected malignancies associated with MF is important. To help identify the common illnesses associated with MF, a cross sectional study was conducted to evaluate 580 adult patients with diagnosed MF.

## 2. Materials and Methods 

We performed a cross-sectional study of patients age 18 and older treated at Johns Hopkins Hospital System (JHHS) between January 1, 2013 and January 1, 2019. Johns Hopkins is a tertiary care referral center with a diverse catchment area which includes local, regional, national and international patients. Anonymous aggregate-level data was collected therefore institutional review board approval was waved. Lifetime incidences of comorbidities were collected using the electronic medical records system EPIC [10,11,12].

Patients diagnosed with MF were compared with three groups: all adults who presented to JHHS with diagnoses other than MF (labelled “general population” for the purpose of this study), adult patients with a diagnosis of atopic dermatitis (AD) and adults diagnosed with psoriasis. The list of comorbidities was obtained from the 2015 Medicare report of the most common chronic medical illnesses affecting the United States population [13]. Malignancies previously reported or suspected to be associated with MF such as Hodgkin’s disease, malignant melanoma and cancers of the lung, breast or colon, were also included for analysis. 

Odds ratios, *p*-values and 95% confidence intervals were calculated using chi-squared statistics with one degree of freedom. *p*-values for comparisons of odds ratios were calculated with Z-tests. A Bonferroni-corrected *p*-value of <0.001 was applied to all assessments of statistical significance. Additionally, subgroup analyses stratified by race were performed for the aforementioned comorbidities and malignancies. 

## 3. Results

**Figure 1:**
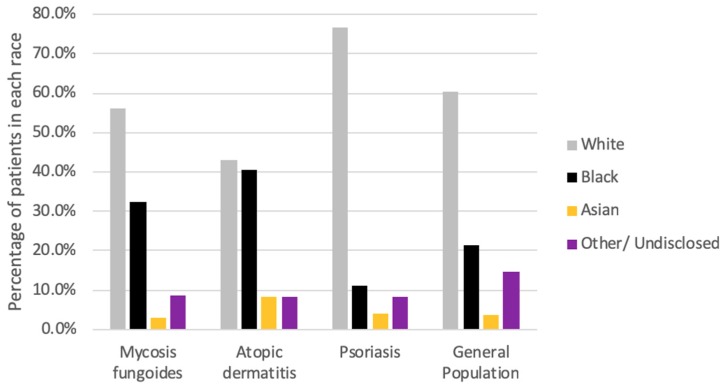
Racial backgrounds of all patients 18 years and older with a diagnosis of mycosis fungoides (MF), atopic dermatitis (AD), or psoriasis and within the general population who presented to the Johns Hopkins Hospital System between January 1, 2013 and January 1, 2019.

**Figure 2:**
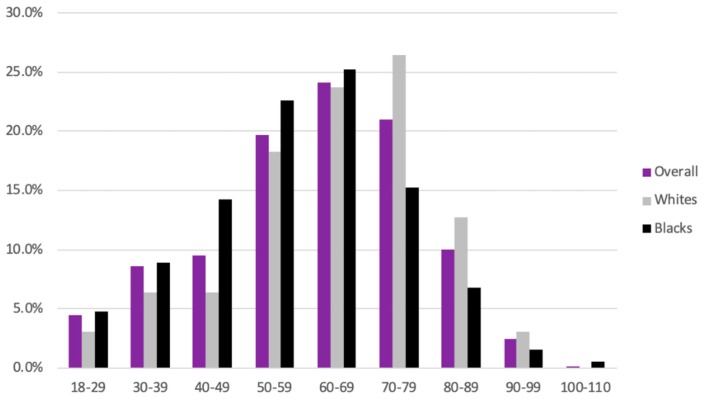
Age distribution of patients with mycosis fungoides (MF) overall, in black and in patients.

Of the 4,944,449 patients that presented to JHHS in the past six years, 580 were diagnosed with MF. Of these, 56.1% were Caucasian, 32.4% were African American and 2.9% were Asian. (Figure 1) Overall, the majority (45.1%) of the MF patients were between the ages of 60 to 79 years. However, the African American MF patients were, on average, younger than the Caucasian group—47.9% of the African Americans belonged to the 50 to 69-year group while 50.1% of Caucasians belonged to the 60 to 79-year group (Figure 2).

Overall (inclusive of all races), MF was statistically significantly associated with certain cutaneous and systemic conditions when compared with all three groups—the general population, patients with AD and patients diagnosed with psoriasis. These included major depressive disorder, Hodgkin’s disease, lymphomatoid papulosis (*p* < 0.001 vs. all control groups). Patients with MF were also significantly less likely to have vitamin D deficiency (*p* < 0.001 vs. all control groups) (Table A1). 

However, on stratifying by race there were important differences in the associated comorbidities. Among the Caucasian study group there was a statistically significant association with lymphomatoid papulosis (*p* < 0.001 vs. all control groups). However, no instances of lymphomatoid papulosis were observed among African American patients with MF. Additionally, on racial sub stratification the association with some conditions was lost; these included congestive heart failure and atopic dermatitis (Table A2 and Table A3).

Compared with psoriasis, MF was not associated with chronic obstructive pulmonary disease (COPD), ischemic heart disease, atrial fibrillation, allergic contact dermatitis, venous thrombosis, chronic kidney disease, malignant melanoma or Alzheimer’s disease. Compared with AD, MF was not associated with osteoarthritis, rheumatoid arthritis, hyperlipidemia, diabetes mellitus type 2 and psoriasis. There was no association found with chronic hepatitis C, inflammatory bowel disease, HIV, Autistic disorder, schizophrenia, ischemic stroke and lung, breast or colon cancer on comparison with AD, psoriasis and/or the general population. The number of Asian patients in our MF cohort was too small to determine associated conditions (Table A2 and Table A3).

## 4. Discussion

This study is among the few analyses of dermatoses, malignancies and comorbidities associated with MF. In line with the literature, the majority (64.8%) of our study group were aged between 50 and 79 years [2,3]. However, in contrast to previous studies the number of females in our MF cohort outnumbered the males (51% vs. 49%) [2,3,14,15,16]. Concordant with earlier observations, we found that Caucasians were most commonly affected, constituting 56.1% of our patient cohort [2,8,17]. However, African Americans were disproportionately affected as compared to the general population; about 32.4% of the patients diagnosed with MF in our study were African American, whereas only 21.3% of all patients seen at JHHS over the same six-year period were African American (Figure 1). Indeed, some earlier studies have found higher incidence rates of MF in African Americans than Caucasians [3,18,19]. Our African American cohort also tended to be younger than the Caucasian group (Figure 2). These are similar to the observations of Huang et al. and are important since African Americans with MF have been found to have significantly shorter overall survival when compared with age at onset-, stage- and treatment-matched Caucasian patients [17,20].

Our study found that MF in Caucasian patients is statistically significantly associated with lymphomatoid papulosis, a finding that was not seen in our African American cohort. White patients with MF were 2147.0 times more likely to have lymphomatoid papulosis than Caucasians in the general population (odds ratio (OR) 2147.0; 95% CI 1062.6–4338.1) and 47.5 times more likely to have lymphomatoid papulosis than race-matched atopic dermatitis patients (OR 47.5; 95% CI 13.0–173.3) (Table A2 and Table A3). Although, the association of MF with lymphomatoid papulosis is well known, this racial difference is significant [21]. In both our African American as well as our Caucasian study groups, MF disease was found to be statistically significantly associated with Hodgkin’s disease. This confirms reports of previous studies that found an association between MF and neoplastic disorders like lymphomatoid papulosis and Hodgkin’s disease [22,23,24,25,26]. The possible reasons for an increased risk of developing second malignancies in MF patients could either be due to the treatment used or similar dysfunctional immune surveillance leading to clonal proliferation [24,27,28]. Alternatively, the two neoplasms may have common genetic events and originator cells contributing to the development of different clones [26]. Considering the high odds of lymphomatoid papulosis and Hodgkin’s disease being associated with MF, long term monitoring of MF patients is prudent regardless of the treatment protocol used. However, the significant racial difference observed suggest monitoring for development of MF may be more beneficial in Caucasian compared to African American patients with lymphomatoid papulosis. 

Whether atopic dermatitis contributes to the development of MF has remained controversial. Results from previous studies have indicated a modest increase in the risk of lymphoma in patients with AD [11]. We found that overall (inclusive of all races), patients with MF were not significantly more likely to have AD when compared with patients with psoriasis (*p* = 0.022). Moreover, this was corroborated when racial sub-group analysis was performed. One possible explanation for the observations in earlier studies is early misclassification of some MF cases as AD. Due to similarities in clinical presentation, there can be a delay from the date of presentation to the diagnosis of MF. A recent study observed higher rates of pruritus associated with malignancies, particularly in case of cutaneous lymphoma in blacks which may add to the difficulty in initial diagnosis [29]. Also, often long term or treatment refractory cases labelled AD may get re-biopsied and demonstrate MF. 

A recent retrospective analysis demonstrated that about 12.7% (41 of 321) of the MF patients had associated psoriasis; of these, 20 patients had psoriasis coexistent with MF. The authors of this study suggested that this association was less likely due to misclassification and could be due to the underlying abnormal T-cell activation in psoriasis which can potentially contribute to the development of cancer [5]. However, contrary to their observations, we found that when compared with patients with AD, MF patients were not significantly more likely to have psoriasis (OR 1.1; CI 0.7–1.7; *p* = 0.6), suggesting that misclassification contributes to an inflated number of psoriasis cases thought to be associated with MF. Nevertheless, further investigation into this may be warranted. 

Vitamin D deficiency has earlier been thought to play a role in triggering MF [30]. In contrast, our study found that MF patients (all races) were 4.6 times (*p* < 0.001) significantly less likely to have vitamin D deficiency than the general population. This is maintained on subgroup analysis: Caucasian MF patients were significantly less likely to have concomitant vitamin D deficiency compared with AD, psoriasis and general population (*p* < 0.001). Our African American cohort was also significantly less likely to have vitamin D deficiency when compared with the general population (Table A2). These observations diverge from that of Talpur et al. who found about 77% of their CTCL patients to be deficient in vitamin D deficiency [31]. One rationale could be the use of phototherapy which may have led to improved vitamin D levels in our cohort, however, further longitudinal studies may be required for confirmation.

Interestingly, our data identified a statistically significant association with congestive heart failure, compared with the general population and AD. This association was maintained on sub stratification by race—both Caucasian and African American patients had a significant association with congestive heart failure. Also, there was a significant association with important coronary artery disease (CAD) risk factors, hypertension and hyperlipidemia (OR 5.3 and 5.0, respectively; *p* < 0.001), when compared with the general population. The association was comparable on racial sub-group analysis (Table A1, Table A2 and Table A3). A possible explanation for this could be that acquired cardiovascular diseases as well as common coronary artery disease (CAD) risk factors (hypertension and hyperlipidemia) are more frequent beyond middle age which is the most commonly affected age group in MF [32]. However, Cengiz et al. recently demonstrated an increased rate of cardiovascular risk factors (hyperlipidemia, high homocysteine and high-sensitivity C-reactive protein) in a cohort of MF patients who did not have pre-existing metabolic disease and were lifetime non-smokers, suggesting a real increase in cardiovascular morbidity by virtue of MF [7]. This begs the chicken or the egg dilemma: are these patients at risk for developing the risk factors for cardiovascular disease (CVD), or do they also have an increased risk of CVD independent of known changes in risk factors or perhaps MF exacerbates risk factors. Further examination of cardiovascular disease and CAD risk factors in MF is suggested by these findings. However, when compared with psoriasis the association of MF with important CAD risk factors, hypertension and hyperlipidemia, was significantly less likely (*p* < 0.001). This held true for all sub-group analyses except in the case of hyperlipidemia in African Americans (Table A1, Table A2 and Table A3). 

Our study has several limitations. First, its cross-sectional design prohibits determining a temporal relationship. The data was representative of the population seen at a single tertiary care hospital system in the United States and thus, may not be generalizable. Also, misclassification of early MF as morphologically similar dermatoses like atopic dermatitis, contact dermatitis and psoriasis is possible and could potentially contribute to an inflated percentage of these dermatoses showing up in our results. Additionally, the comparison group “general population” in our study refers to the total population excluding patients with MF, however, since patients with AD and psoriasis are included in this group it may lead to an overestimation or underestimation of results. Moreover, as aggregate level data was used, gender information missing at the time of analysis may account for our unexpected sex distribution. Finally, there may be additional unknown confounding factors such as socioeconomic status or preexisting medical comorbidities in the mycosis fungoides group that could have affected the interpretation of our results.

## 5. Conclusions

These results indicate that MF is strongly associated with Hodgkin’s disease, and that MF in Caucasians is associated with lymphomatoid papulosis. This finding in this study suggests that clinicians consider long term follow up for Caucasian but not African American patients with lymphomatoid papulosis to monitor for development of MF. Moreover, we found that although MF is significantly associated with CAD risk factors compared with the general population, these associations were less likely on comparison with psoriasis. On comparison with clinical mimickers, MF was not significantly associated with contact dermatitis (compared with psoriasis) and psoriasis (compared with AD) or lung, breast and colon cancer, conditions that have previously been thought to be associated with MF.

## Appendix A

**Table A1:** Absolute number, percentage, odds ratios and *p* values of all patients 18 years and older with MF and various comorbid conditions, as compared with those of patients 18 and older with AD, with psoriasis, or within the general population (without MF) who were seen at the Johns Hopkins Hospital System (JHHS) between January 1, 2013 and January 1, 2019. Data presented for 580 patients with mycosis fungoides (MF), 10,382 patients with AD, 15,051 patients with psoriasis, and 4,943,869 patients in the general population (excluding those with MF). AD, Atopic dermatitis; COPD, chronic obstructive pulmonary disease; OR, odds ratio; PN, prurigo nodularis.

Comorbidity	MF, n (%)	AD, n (%)	OR (95% CI)	*p* Value	Psoriasis, n (%)	OR (95% CI)	*p* Value	Gen Pop, n (%)	OR (95% CI)	*p* Value
Allergic Contact Dermatitis	6 (1.0)	428 (4.1)	0.2 (0.1–0.5)	<0.001	137 (0.9)	1.1 (0.5–2.6)	0.75779	4876 (0.1)	10.6 (4.7–23.7)	<0.001
Alzheimer Disease	5 (0.9)	27 (0.3)	3.3 (1.3–8.7)	0.00892	105 (0.7)	1.2 (0.5–3.0)	0.64202	8882 (0.2)	4.8 (2.0–11.7)	<0.001
Asthma	38 (6.6)	2223 (21.4)	0.3 (0.2–0.4)	<0.001	1529 (10.2)	0.6 (0.4–0.9)	0.00454	109,109 (2.2)	3.1 (2.2–4.3)	<0.001
Atopic Dermatitis	23 (4.0)				369 (2.5)	1.6 (1.1–2.5)	0.02214	10,358 (0.2)	19.7 (13.0–29.9)	<0.001
Atrial Fibrillation	32 (5.5)	284 (2.7)	2.1 (1.4–3.0)	<0.001	931 (6.2)	0.9 (0.6–1.3)	0.51122	68,394 (1.4)	4.2 (2.9–5.9)	<0.001
Autistic Disorder	0 (0)	50 (0.5)	0	0.09391	19 (0.1)	0	0.39189	2371 (0.0)	0	0.59782
Breast Cancer	2 (0.3)	33 (0.3)	1.1 (0.3–4.5)	0.91079	76 (0.5)	0.7 (0.2–2.8)	0.59126	7509 (0.2)	2.3 (0.6–9.1)	0.23285
Chronic Hepatitis C	3 (0.5)	130 (1.3)	0.4 (0.1–1.3)	0.11564	214 (1.4)	0.4 (0.1–1.1)	0.06769	13,952 (0.3)	1.8 (0.6–5.7)	0.28601
Chronic Kidney Disease	41 (7.1)	446 (4.3)	1.7 (1.2–2.4)	0.00161	950 (6.3)	1.1 (0.8–1.6)	0.46281	57,386 (1.2)	6.5 (4.7–8.9)	<0.001
Colon Cancer	0 (0)	8 (0.1)	0	0.50364	27 (0.2)	0	0.3073	2561 (0.1)	0	0.5835
Congestive Heart Failure	37 (6.4)	287 (2.8)	2.4 (1.7–3.4)	<0.001	675 (4.5)	1.5 (1.0–2.0)	0.03177	43,133 (0.9)	7.7 (5.5–10.8)	<0.001
COPD	36 (6.2)	424 (4.1)	1.6 (1.1–2.2)	0.01308	961 (6.4)	1.0 (0.7–1.4)	0.86328	51,410 (1.0)	6.3 (4.5–8.8)	<0.001
Diabetes Mellitus Type 2	64 (11.0)	103 (9.7)	1.2 (0.9–1.5)	0.27746	2191 (14.6)	0.7 (0.6–0.9)	0.01782	136,538 (2.8)	4.4 (3.4–5.7)	<0.001
HIV	3 (0.5)	185 (1.8)	0.3 (0.1–0.9)	0.02243	144 (1.0)	0.5 (0.4–0.7)	0.28188	13,283 (0.3)	1.9 (0.6–6.0)	0.24755
Hodgkin’s Disease	7 (1.2)	10 (0.1)	12.7 (4.8–33.4)	<0.001	20 (0.1)	9.2 (3.9–21.8)	<0.001	1631 (0.0)	37.0 (17.5–78.1)	<0.001
Hyperlipidemia	155 (26.7)	2736 (26.4)	1.0 (0.8–1.2)	0.84364	5,812 (38.6)	0.6 (0.5–0.7)	<0.001	335,534 (6.8)	5.0 (4.2–6.0)	<0.001
Hypertension	194 (33.4)	2991 (28.8)	1.2 (1.0–1.5)	0.01664	6128 (40.7)	0.7 (0.6–0.9)	<0.001	427,318 (8.6)	5.3 (4.5–6.3)	<0.001
Inflammatory Bowel Disease	5 (0.9)	94 (0.9)	1.0 (0.4–2.3)	0.91448	228 (1.5)	0.6 (0.2–1.4)	0.203	8817 (0.2)	4.9 (2.0–11.7)	<0.001
Ischemic Heart Disease	53 (9.1)	512 (4.9)	1.9 (1.4–2.6)	<0.001	1274 (8.5)	1.1 (0.8–1.5)	0.56805	79,665 (1.6)	6.1 (4.6–8.1)	<0.001
Ischemic Stroke	0 (0)	23 (0.2)	0	0.25649	37 (0.2)	0	0.23189	2715 (0.1)	0	0.5724
Lung Cancer	3 (0.5)	30 (0.3)	1.8 (0.5–5.9)	0.32876	83 (0.6)	0.9 (0.3–3.0)	0.91295	7229 (0.1)	3.6 (1.1–11.0)	0.01939
Lymphomatoid Papulosis	11 (1.9)	4 (0.0)	50.2 (15.9–158)	<0.001	-	0	<0.001	49 (0.0)	1950.5 (1009.0–3770.4)	<0.001
Major Depressive Disorder	44 (7.6)	1340 (12.9)	0.6 (0.4–0.8)	<0.001	2091 (13.9)	0.5 (0.4–0.7)	<0.001	119,308 (2.4)	3.3 (2.4–4.5)	<0.001
Malignant Melanoma	9 (1.6)	70 (0.7)	2.3 (1.2–4.7)	0.01504	164 (1.1)	1.4 (0.7–2.8)	0.29657	7879 (0.2)	9.9 (5.1–19.1)	<0.001
Osteoarthritis	80 (13.8)	1689 (16.3)	0.8 (0.6–1.0)	0.11477	3981 (26.5)	0.4 (0.4–0.6)	<0.001	187,109 (3.8)	4.1 (3.2–5.2)	<0.001
Osteoporosis	13 (2.2)	417 (4.0)	0.5 (0.3–1.0)	0.0321	870 (5.8)	0.4 (0.2–0.7)	<0.001	44,238 (0.9)	2.5 (1.4–4.4)	<0.001
Psoriasis	23 (4.0)	369 (3.6)	1.1 (0.7–1.7)	0.60368				15,028 (0.3)	13.5 (8.9–20.6)	<0.001
Rheumatoid Arthritis	8 (1.4)	128 (1.2)	1.1 (0.5–2.3)	0.75656	493 (3.3)	0.4 (0.2–0.8)	0.01095	11,071 (0.2)	6.2 (3.1–12.5)	<0.001
Schizophrenia	2 (0.3)	73 (0.7)	0.5 (0.1–2.0)	0.30831	71 (0.5)	0.7 (0.2–3.0)	0.66003	8507 (0.2)	2.0 (0.5–8.0)	0.31551
Venous Thrombosis	26 (4.5)	268 (2.6)	1.8 (1.2-2.7)	0.00581	496 (3.3)	1.4 (0.9-2.1)	0.11836	34,113 (0.7)	6.8 (4.6–10.0)	<0.001
Vitamin D Deficiency	65 (11.2)	2052 (19.8)	0.5 (0.4–0.7)	<0.001	2996 (19.9)	0.5 (0.4–0.7)	<0.001	132,446 (2.7)	4.6 (3.5–5.9)	<0.001

**Table A2:** Absolute number, percentage, odds ratios and *p* values of Black/African American patients age 18 years and older with mycosis fungoides and various comorbid conditions, as compared with race-matched controls age 18 years and older with AD, with psoriasis, or within the general population who were seen at the JHHS between January 1, 2013 and January 1, 2019. Data presented for 190 patients with mycosis fungoides (MF), 4287 patients with AD, 1690 patients with psoriasis, and 944,278 patients in the general population (excluding those with MF). AD, Atopic dermatitis; COPD, chronic obstructive pulmonary disease; OR, odds ratio; PN, prurigo nodularis; IBS, inflammatory bowel disease.

Comorbidity	MF, n (%)	AD, n (%)	OR (95% CI)	*p* Value	Psoriasis, n (%)	OR (95% CI)	*p* Value	Gen Pop, n (%)	OR (95% CI)	*p* Value
Allergic Contact Dermatitis	2 (1.1)	118 (2.8)	0.4 (0.1–1.5)	0.15571	22 (1.3)	0.8 (0.2–3.5)	0.77179	893 (0.1)	11.2 (2.8–45.3)	<0.001
Alzheimer Disease	2 (1.1)	10 (0.2)	4.6 (1.0–20.9)	0.03255	11 (0.7)	1.6 (0.4–7.4)	0.52635	1742 (0.2)	5.8 (1.4–23.2)	0.00532
Asthma	18 (9.5)	1240 (28.9)	0.3 (0.2–0.4)	<0.001	266 (15.7)	0.6 (0.3–0.9)	0.02221	40,063 (4.2)	2.4 (1.4–3.8)	<0.001
Atopic Dermatitis	11 (5.8)				93 (5.5)	1.1 (0.6–2.0)	0.86989	4276 (0.5)	13.5 (7.3–24.9)	<0.001
Atrial Fibrillation	13 (6.8)	74 (1.7)	4.2 (2.3–7.7)	<0.001	75 (4.4)	1.6 (0.9–2.9)	0.13687	10,168 (1.1)	6.7 (3.8–11.9)	<0.001
Autistic Disorder		17 (0.4)	0	0.38448	6 (0.4)	0	0.41072	687 (0.1)	0	0.70994
Breast Cancer	2 (1.1)	13 (0.3)	3.5 (0.8–15.6)	0.08025	10 (0.6)	1.8 (0.4–8.2)	0.44942	1465 (0.2)	6.8 (1.7–27.6)	0.00168
Chronic Hepatitis C	2 (1.1)	95 (2.2)	0.5 (0.1–1.9)	0.28112	64 (3.8)	0.3 (0.1–1.1)	0.05218	7057 (0.7)	1.4 (0.4–5.7)	0.62517
Chronic Kidney Disease	18 (9.5)	224 (5.2)	1.9 (1.1–3.1)	0.01127	179 (10.6)	0.9 (0.5–1.5)	0.63332	20,616 (2.2)	4.7 (2.9–7.6)	<0.001
Colon Cancer		1 (0.0)	0	0.83324	2 (0.1)	0	0.63519	461 (0.0)	0	0.76064
Congestive Heart Failure	17 (8.9)	136 (3.2)	3.0 (1.8–5.1)	<0.001	115 (6.8)	1.3 (0.8–2.3)	0.2731	13,103 (1.4)	7.0 (4.2–11.5)	<0.001
COPD	13 (6.8)	195 (4.5)	1.5 (0.9–2.8)	0.14163	120 (7.1)	1.0 (0.5–1.7)	0.89518	13,161 (1.4)	5.2 (3.0–9.1)	<0.001
Diabetes Mellitus Type 2	34 (17.9)	525 (12.2)	1.6 (1.1–2.3)	0.02118	379 (22.4)	0.8 (0.5–1.1)	0.15263	47,086 (50.)	4.2 (2.9–6.0)	<0.001
HIV	3 (1.6)	158 (3.7)	0.4 (0.1–1.3)	0.12699	75 (4.4)	0.3 (0.1–1.1)	0.06099	8937 (0.9)	1.7 (0.5–5.3)	0.36796
Hodgkin’s Disease	2 (1.1)	2 (0.0)	22.8 (3.2–162.7)	<0.001	2 (0.1)	9.0 (1.3–64.1)	0.00805	273 (0.0)	36.8 (9.1–148.9)	<0.001
Hyperlipidemia	48 (25.3)	961 (22.4)	1.2 (0.8–1.6)	0.35813	632 (37.4)	0.6 (0.4–0.8)	<0.001	72,033 (7.6)	4.1 (3.0–5.7)	<0.001
Hypertension	81 (42.6)	1333 (31.1)	1.6 (1.2–2.2)	<0.001	884 (52.3)	0.7 (0.5–0.9)	0.01141	127,879 (13.5)	4.7 (3.6–6.3)	<0.001
IBS	2 (1.1)	28 (0.7)	1.6 (0.4–6.8)	0.50894	18 (1.1)	1.0 (0.2–4.3)	0.98734	1363 (0.1)	7.4 (1.8–29.7)	<0.001
Ischemic Heart Disease	20 (10.5)	243 (5.7)	2.0 (1.2–3.2)	0.00533	183 (10.8)	1.0 (0.6–1.6)	0.89878	21,833 (2.3)	5.0 (3.1–7.9)	<0.001
Ischemic Stroke		7 (0.2)	0	0.57723	6 (0.4)	0	0.41072	847 (0.1)	0	0.6796
Lung Cancer	1 (0.5)	8 (0.2)	2.8 (0.4–22.7)	0.30631	14 (0.8)	0.6 (0.1–4.8)	0.65722	1321 (0.1)	3.8 (0.5–27.0)	0.15429
Lymphomatoid Papulosis		1 (0.0)	0	0.83324				7 (0.0)	0	0.97006
Major Depressive Disorder	18 (9.5)	615 (14.3)	0.6 (0.4–1.0)	0.05928	265 (15.7)	0.6 (0.3–0.9)	0.0233	29,514 (3.1)	3.2 (2.0–5.3)	<0.001
Malignant Melanoma		3 (0.1)	0	0.71529				177 (0.0)	0	0.8503
Osteoarthritis	28 (14.7)	643 (15.0)	1.0 (0.7–1.5)	0.92114	433 (25.6)	0.5 (0.3–0.8)	<0.001	44,608 (4.7)	3.5 (2.3–5.2)	<0.001
Osteoporosis	2 (1.1)	83 (1.9)	0.5 (0.1–2.2)	0.38258	75 (4.4)	0.2 (0.1–0.9)	0.0256	5538 (0.6)	1.8 (0.4–7.3)	0.40015
Psoriasis	9 (4.7)	93 (2.2)	2.2 (1.1–4.5)	0.02029				1681 (0.2)	27.9 (14.2–54.5)	<0.001
Rheumatoid Arthritis	3 (1.6)	54 (1.3)	1.3 (0.4–4.1)	0.70085	60 (3.6)	0.4 (0.1–1.4)	0.15226	13,171 (1.4)	1.1 (0.4–3.5)	0.82869
Schizophrenia		53 (1.2)	0	0.12313	24 (1.4)	0	0.09829	5083 (0.5)	0	0.31056
Venous Thrombosis	10 (5.3)	114 (2.7)	2.0 (1.0–3.9)	0.03233	74 (4.4)	1.2 (0.6–2.4)	0.57583	9077 (1.)	5.7 (3.0–10.8)	<0.001
Vitamin D Deficiency	30 (15.8)	882 (20.6)	0.7 (0.5–1.1)	0.10909	433 (25.6)	0.5 (0.4–0.8)	0.00286	38,158 (4.0)	4.5 (3.0–6.6)	<0.001

**Table A3:** Absolute number, percentage, odds ratios and *p* values of White/Caucasian patients age 18 years and older with mycosis fungoides (MF) and various comorbid conditions, as compared with race-matched controls age 18 years and older with AD, with psoriasis, or within the general population who were seen at the JHHS between January 1, 2013 and January 1, 2019. Data presented for 329 patients with mycosis fungoides (MF), 4545 patients with AD, 11,700 patients with psoriasis, and 2,671,135 patients in the general population (excluding those with MF). AD, Atopic dermatitis; COPD, chronic obstructive pulmonary disease; OR, odds ratio; PN, prurigo nodularis; IBS, inflammatory bowel disease.

Comorbidity	MF, n (%)	AD, n (%)	OR (95% CI)	*p* Value	Psoriasis, n (%)	OR (95% CI)2	*p* Value	Gen Pop, n (%)	OR (95% CI)	*p* Value
Allergic Contact Dermatitis	4 (1.2)	249 (5.5)	0.2 (0.1–0.6)	<0.001	100 (0.9)	1.4 (0.5–3.9)	0.48534	3294 (0.1)	10.0 (3.7–26.7)	<0.001
Alzheimer Disease	3 (0.9)	15 (0.3)	2.8 (0.8–9.6)	0.09295	88 (0.8)	1.2 (0.4–3.9)	0.7416	6306 (0.2)	3.9 (1.2–12.1)	0.01157
Asthma	19 (5.8)	764 (16.8)	0.3 (0.2–0.5)	<0.001	1138 (9.7)	0.6 (0.4–0.9)	0.01651	58,241 (2.2)	2.7(1.7–4.4)	<0.001
Atopic Dermatitis	11 (3.3)				224 (1.9)	1.8 (1.0–3.3)	0.06476	4534 (0.2)	20.3 (11.1–37.1)	<0.001
Atrial Fibrillation	18 (5.5)	187 (4.1)	1.3 (0.8–2.2)	0.23646	808 (6.9)	0.8 (0.5–1.3)	0.31012	53,118 (2.0)	2.9 (1.8–4.6)	<0.001
Autistic Disorder		29 (0.6)	0	0.14617	10 (0.1)	0	0.59577	1451 (0.1)	0	0.67239
Breast Cancer		18 (0.4)	0	0.25279	62 (0.5)	0	0.18557	4966 (0.2)	0	0.43374
Chronic Hepatitis C	1 (0.3)	29 (0.6)	0.5 (0.1–3.5)	0.45432	137 (1.2)	0.3 (0.0–1.8)	0.14529	6140 (0.2)	1.3 (0.2–9.4)	0.7789
Chronic Kidney Disease	20 (6.1)	187 (4.1)	1.5 (0.9–2.4)	0.08793	707 (6.0)	1.0 (0.6–1.6)	0.97826	31,639 (1.2)	5.4 (3.4–8.5)	<0.001
Colon Cancer		4 (0.1)	0	0.59036	23 (0.2)	0	0.42083	1842 (0.1)	0	0.63373
Congestive Heart Failure	18 (5.5)	132 (2.9)	1.9 (1.2–3.2)	0.00924	518 (4.4)	1.2 (0.8–2.0)	0.3655	26,637 (1.0)	5.7 (3.6–9.2)	<0.001
COPD	21 (6.4)	209 (4.6)	1.4 (0.9–2.2)	0.14046	797 (6.8)	0.9 (0.6–1.5)	0.7605	35,482 (1.3)	5.1 (3.3–7.9)	<0.001
Diabetes Mellitus Type 2	24 (7.3)	346 (7.6)	1.0 (0.6–1.5)	0.83347	1562 (13.4)	0.5 (0.3–0.8)	0.00137	70,060 (2.6)	2.9 (1.9–4.4)	<0.001
HIV		18 (0.4)	0	0.25279	63 (0.5)	0	0.18204	3463 (0.1)	0	0.51342
Hodgkin’s Disease	4 (1.2)	5 (0.1)	11.2 (3.0–41.8)	<0.001	16 (0.1)	9.0 (3.0–27.0)	<0.001	1103 (0.0)	29.8 (11.1–80.0)	<0.001
Hyperlipidemia	99 (30.1)	1408 (31.0)	1.0 (0.8–1.2)	0.73649	4631 (39.6)	0.7 (0.5–0.8)	<0.001	221,792 (8.3)	4.8 (3.8–6.0)	<0.001
Hypertension	102 (31.0)	1337 (29.4)	1.1 (0.8–1.4)	0.5425	4733 (40.5)	0.7 (0.5–0.8)	<0.001	251,808 (9.4)	4.3 (3.4–5.5)	<0.001
IBS	2 (0.6)	50 (1.1)	0.5 (0.1–2.3)	0.40138	198 (1.7)	0.4 (0.1–1.4)	0.12925	6582 (0.2)	2.5 (0.6–9.9)	0.18606
Ischemic Heart Disease	29 (8.8)	224 (4.9)	1.9 (1.2–2.8)	0.00215	995 (8.5)	1.0 (0.7–1.5)	0.84233	50,371 (1.9)	5.0 (3.4–7.4)	<0.001
Ischemic Stroke	-	14 (0.3)	0	0.31339	28 (0.2)	0	0.37435	1564 (0.1)	0	0.66064
Lung Cancer	2 (0.6)	16 (0.4)	1.7 (0.4–7.6)	0.46001	65 (0.6)	1.1 (0.3–4.5)	0.89987	5063 (0.2)	3.2 (0.8–12.9)	0.08111
Lymphomatoid Papulosis	10 (3.0)	3 (0.1)	47.5 (13.0-173.3)	<0.001				39 (0.0)	2147.0 (1062.6–4338.1)	<0.001
Major Depressive Disorder	25 (7.6)	617 (13.6)	0.5 (0.3–0.8)	0.00197	1720 (14.7)	0.5 (0.3–0.7)	<0.001	80,063 (3.0)	2.7 (1.8–4.0)	<0.001
Malignant Melanoma	9 (2.7)	65 (1.4)	1.9 (1.0–3.9)	0.0615	157 (1.3)	2.1 (1.0–4.1)	0.03259	7314 (0.3)	10.2 (3.7–26.7)	<0.001
Osteoarthritis	48 (14.6)	869 (19.1)	0.7 (0.5–1.0)	0.04233	3281 (28.0)	0.4 (0.3–0.6)	<0.001	125,515 (4.7)	3.5 (2.6–4.7)	<0.001
Osteoporosis	10 (3.0)	261 (5.7)	0.5 (0.3–1.0)	0.03882	729 (6.2)	0.5 (0.3–0.9)	0.01744	33,605 (1.3)	2.5 (1.3–4.6)	0.00375
Psoriasis	12 (3.6)	224 (4.9)	0.7 (0.4–1.3)	0.29586				11,689 (0.4)	8.6 (4.8–15.3)	<0.001
Rheumatoid Arthritis	5 (1.5)	58 (1.3)	1.2 (0.5–3.0)	0.70559	390 (3.3)	0.4 (0.2–1.1)	0.06869	35,498 (1.3)	1.1 (0.5–2.8)	0.76249
Schizophrenia	2 (0.6)	16 (0.4)	1.7 (0.4–7.6)	0.46001	43 (0.4)	1.7 (0.4–6.9)	0.4812	2777 (0.1)	5.9 (1.5–23.6)	0.00458
Venous Thrombosis	12 (3.6)	130 (2.9)	1.3 (0.7–2.3)	0.41235	403 (3.4)	1.1 (0.6–1.9)	0.84231	22,078 (0.8)	4.5 (2.6–8.1)	<0.001
Vitamin D Deficiency	29 (8.8)	838 (18.4)	0.4 (0.3–0.6)	<0.001	2259 (19.3)	0.4 (0.3–0.6)	<0.001	75,560 (2.8)	3.3 (2.3–4.9)	<0.001
